# Quantität und Qualität des Strategieeinsatzes von Grundschülerinnen und Grundschülern in reziproken Lesegruppen

**DOI:** 10.1007/s42278-022-00142-1

**Published:** 2022-05-24

**Authors:** Vanessa A. Völlinger, Lisa-Kristin Stein, Agnes Eckart

**Affiliations:** Pädagogisch-Psychologische Interventionsforschung, Fachbereich Psychologie und Sportwissenschaft, Justus-Liebig-Universität Gießen, Gießen, Deutschland

**Keywords:** Lesestrategien, Leseförderung, Video-Analyse, Gruppenarbeit, Reading strategies, Fostering reading, Video analysis, Group work

## Abstract

Zur Förderung der Leseleistungen von Schülerinnen und Schülern werden von nationalen und internationalen Experten Programme empfohlen, in denen Kinder gemeinsam an Leseaufgaben arbeiten und verschiedene Lesestrategien erlernen. Die Ergebnisse von Evaluationsstudien belegen die Wirksamkeit solcher Programme für die Entwicklung individueller Leseleistungen von Schülerinnen und Schülern, enthalten jedoch keine Informationen dazu, wie die gemeinsame Strategieanwendung in den Kleingruppen abläuft. Der vorliegende Beitrag betrachtet anhand von Videomaterial zu drei Messzeitpunkten, ob und wie Schülerinnen und Schüler der 3. Klasse gemeinsam die Lesestrategien *Klären, Fragen *und *Vorhersagen *auf Sachtexte anwenden und ob ein Trainingseffekt für Kinder in Kleingruppen festzustellen ist, die an einer peer-gestützten strategiebasierten Leseförderung teilgenommen haben*. *Dazu wird eine Interventionsgruppe mit einer Unterrichtskontrollgruppe verglichen, die regulären Deutschunterricht ohne explizite Strategieförderung erhielt. Sowohl die Quantität als auch die Qualität der Strategieanwendung in den Lesegruppen wurde anhand eines deduktiv entwickelten Schemas kodiert. Die Ergebnisse zeigen, dass zu allen drei Messzeitpunkten (Prä‑, Post- und Follow-up-Test) weniger als 50 % der Schülerinnen und Schüler Lesestrategien anwendeten. Am häufigsten wurde insgesamt die Strategie *Fragen *genutzt. Ein moderat positiver Trainingseffekt zeigte sich insbesondere für die Strategie *Vorhersagen*. Zu diskutieren ist, warum die Schülerinnen und Schüler nicht mehr Strategien auf einem höheren Elaborationsniveau anwendeten und ob weitere Kompetenzen für die wirkungsvolle Strategieanwendung in Kleingruppen benötigt werden. Die Untersuchung unterstreicht die Bedeutung prozessnaher Datenerhebung für die Weiterentwicklung von Fördermaßnahmen in Forschung und Praxis.

**Zusatzmaterial online:**

Zusätzliche Informationen sind in der Online-Version dieses Artikels (10.1007/s42278-022-00142-1) enthalten.

## Abstract (en)

To promote students’ reading achievement, national and international experts recommend programs in which children collaborate on reading tasks and learn various reading strategies. The results of evaluation studies show the effectiveness of such programs for the development of individual reading performance of students, but do not contain any information on how the joint strategy application takes place in the small groups. Using video data at three measurement time points, this paper examines whether and how 3rd grade students cooperatively apply the strategies of *clarifying, questioning *and *predicting *to nonfiction texts. Therefore, an intervention group that participated in peer-supported strategy-based reading instruction was compared to a control group that received regular German lessons without explicit strategy support. Both the quantity and quality of strategy use in the reading groups was coded using a deductively developed scheme. Results show that at all three measurement time points (pre-test, post-test, and follow-up), less than 50% of the students used reading strategies. Overall, the strategy most frequently used was *questioning*. A moderate positive training effect was particularly evident for *predicting*. What needs to be discussed is why students did not use more strategies at a higher level of elaboration and whether additional skills are needed for effective strategy use in small groups. The study underscores the importance of process-based data collection for the continued development of remedial approaches in research and practice.

## Einleitung

Die Entwicklung der Lesekompetenz ist eines der Hauptziele, die Kinder während ihrer Grundschulzeit erreichen sollen. Aus Erhebungen im Rahmen von Schulleistungsstudien wie der Internationalen Grundschul-Lese-Untersuchung (IGLU; Hußmann et al. 2017) und dem Programme for International Student Assessment (PISA; OECD 2019) ist bekannt, dass sich nicht bei allen Kindern als Konsequenz des Schulunterrichts ein hohes Niveau der Lesekompetenz entwickelt. Insbesondere bei Sachtexten zeigen sich in den Leseleistungen deutscher Schülerinnen und Schüler der 4. Klasse schwächere Verstehensleistungen als in der Vergleichsgruppe der EU-Staaten (Bos et al. 2017).

Dazu kommt, dass laut der Erhebungen im Rahmen von IGLU 2016 der Leseunterricht in deutschen Grundschulklassen im internationalen Vergleich sowohl in der Quantität als auch in der Qualität unterdurchschnittlich abschneidet: Deutsche Lehrkräfte geben an, im Mittel drei Schulstunden pro Woche speziell für Leseunterricht zu verwenden (damit etwa 90 h pro Schuljahr) und liegen mit diesen Angaben deutlich unter dem internationalen Mittelwert (etwa 160 h; Bremerich-Vos et al. 2017). Zudem sind evidenzbasierte Verfahren wie begleitetes (wiederholtes) lautes Lesen zur Förderung der Leseflüssigkeit oder die explizite Instruktion von Lesestrategien nicht Teil alltäglicher Unterrichtspraxis (Bremerich-Vos et al. 2017).

## Förderung verstehenden Lesens durch Lesestrategien und Peer-Unterstützung

Geübte Leserinnen und Leser kontrollieren ihr Textverständnis, indem sie während des Lesens metakognitive und kognitive Strategien anwenden, wie z. B. Vorhersagen, Zusammenfassungen oder die Aktivierung von Vorwissen (Palincsar und Brown 1984). Diese Lesestrategien gehören zu den elaborierten Prozessen, die ein tieferes Textverständnis fördern. Die bedeutende Rolle von Lesestrategien für das Leseverständnis lässt sich sowohl an positiven Trainingseffekten von Interventionen als auch anhand struktureller Modelle darstellen und ablesen. So zeigen Überblicksarbeiten und Metanalysen wiederholt, dass Ansätze zur Förderung von Lesestrategien positive Effekte auf das Leseverständnis haben (Mayer und Marks 2019; Rosenshine und Meister 1994; Souvignier und Antoniou 2007). Förderprogramme, die in den oberen Grundschulklassen ansetzen, erweisen sich als besonders wirksam für die Entwicklung von Wissen und Anwendung von Lesestrategien sowie das Leseverständnis (Mayer und Marks 2019), auch bei lernschwachen Kindern (Souvignier und Antoniou 2007). Die Ergebnisse empirischer Prüfungen von Lesemodellen bestätigen ebenfalls, dass neben Variablen wie Wortschatz, Leseflüssigkeit, Vorwissen oder Lesemotivation auch Lesestrategien bedeutsam zur Vorhersage von Leseverständnisleistungen beitragen (Ahmed et al. 2021). Zudem wirken sie als Mediatoren der Effekte kognitiver und motivationaler Variablen auf das verstehende Lesen (Liao et al. 2021; Völlinger et al. 2018a). Diese Mediationseffekte deuten darauf hin, dass eine gute Strategienutzung nicht isoliert funktioniert, sondern Wortschatzkenntnisse sowie Leseflüssigkeit wichtig sind, um die positive Wirkung von Lesestrategien auf das Leseverständnis zu vermitteln (Völlinger et al. 2018a). Auch kann ein höheres Maß an Lesemotivation zu einer häufigeren Anwendung von Lesestrategien führen, insbesondere von Strategien auf tieferen Ebenen, woraus sich eine bessere Leistung beim Leseverständnis vorhersagen lässt (Liao et al. 2021).

### Evidenzbasierte Lesestrategieprogramme

Empfohlene Programme zur Förderung verstehenden Lesens beinhalten eine Kombination aus Strategievermittlung und Peer-Learning mit zusätzlichen motivationsförderlichen Elementen (Artelt et al. 2007) und stellen damit Multikomponentenprogramme dar. Zentral in diesen Programmen ist jeweils das gemeinsame Üben des flüssigen und betonten Lesens und die Instruktion einer begrenzten Zahl von verständnisfördernden und verständnisprüfenden Lesestrategien. Diese werden gemeinsam in Lesepaaren oder Lesegruppen eingeübt und auf Texte angewendet. Die Lernenden wechseln sich in der Moderation bzw. Überwachung des Leseprozesses ab, wodurch die Metakognition beim Lesen gefördert werden soll (Fuchs und Fuchs 2000; Palincsar und Brown 1984). Diese Elemente finden sich beispielsweise im Reziproken Lehren (RT; Palincsar und Brown 1984), welches ursprünglich für Schülerinnen und Schüler konzipiert wurde, die bereits flüssig jedoch nicht sinnentnehmend lesen konnten. Bei RT erlernen die Schülerinnen und Schüler die Strategien *Klären *unbekannter Wörter und Begriffe, das *Zusammenfassen* zentraler Inhalte von Textabschnitten, das Stellen von* Fragen*, mit denen das Verständnis für den Text überprüft werden kann, und das Treffen von* Vorhersagen* darüber, wie es im Text weitergehen könnte, auf der Grundlage des bereits gelesenen Textes. Eine ähnliche Auswahl an Strategien wird auch im Programm Peer-Assisted Learning Strategies (PALS, Fuchs und Fuchs 2000) vermittelt. Bei PALS arbeiten die Schülerinnen und Schüler in Paaren mit reziprokem Rollentausch zusammen und üben das flüssige Vorlesen von Texten sowie die Strategien Zusammenfassen und Vorhersagen treffen.

Die Frage, welche und wie viele Strategien in solchen Programmen vermittelt werden sollten, ist nicht eindeutig zu beantworten. In der Literatur finden sich positive Trainingseffekte für Programme, in denen eine (z. B. Daniel und Williams 2021), wenige (z. B. Seuring und Spörer 2010) oder eine größere Anzahl an Strategien (z. B. Walter 2020) vermittelt wurden, wobei sich metanalytisch für die Kombination verschiedener Strategien ein Vorteil abzeichnet (Mayer und Marks 2019). Die Anzahl und Auswahl der Strategien sollten an den Umfang der Förderung und das Alter der Teilnehmenden angepasst sein, um insbesondere jüngere Schülerinnen und Schüler nicht zu überfordern (Matthäi und Artelt 2009). Empirische Befunde deuten zudem auf mögliche Interferenzen bei Lernstrategietrainings mit einer höheren Komplexität (Klauer 2010) hin. So berichten Seuring und Spörer (2010) eine höhere Wirksamkeit einer reziproken Leseförderung, in der nur drei Strategien vermittelt wurden (Strategie Zusammenfassen ausgeblendet) im Vergleich zu einer Intervention, in der alle vier Strategien des reziproken Lehrens instruiert wurden.

### Erfassung von Trainingseffekten strategiebasierter Programme

Die umfangreichen Belege für positive Effekte von strategiebasierten Förderprogrammen beziehen sich vor allem auf die Ergebnisse von Lesetests, welche in Evaluationsstudien vor und nach einer Förderung eingesetzt werden (Mayer und Marks 2019). In verschiedenen Untersuchungen wurde auch das Wissen über Lesestrategien oder die Kompetenz in der Anwendung von Lesestrategien in Fragebögen und Testverfahren erhoben und darüber positive Trainingseffekte abgebildet (Mayer und Marks 2019). Allerdings zeigt der Einsatz von retrospektiven Verfahren wie Fragebögen zur Erfassung von Strategien methodische Schwierigkeiten. So konnten in Untersuchungen keine Zusammenhänge zwischen handlungsnah und per Fragebogen erfassten Lesestrategien bei Schülerinnen und Schülern nachgewiesen werden (siehe z. B. Artelt 1999). Als Erklärung für einen fehlenden Zusammenhang wird die Überschätzung des Lesestrategieeinsatzes durch die Kinder selbst herangeführt (Hellmich und Läsche 2009). Hinzu kommt, dass in einer peer-gestützten Lesestrategieförderung die Schülerinnen und Schüler lernen, die Lesestrategien gemeinsam im Gespräch auf einen Text bezogen anzuwenden. Diese Lernsituation und die damit verbundenen Aufgaben der gemeinsamen Strategieanwendung und Gruppenregulation unterscheidet sich demnach deutlich von einer individuellen Befragung oder Testsituation in einem Lesestrategietest, bei dem die Strategieanwendung schriftlich erfasst wird (siehe z. B. Seuring und Spörer 2010).

## Fragestellung

Derzeit gibt es noch keine Informationen dazu, wie die gemeinsame Strategieanwendung in Kleingruppen während des Lesetrainings praktisch durchgeführt wird. Um besser zu verstehen, wie kooperative Leseinterventionen ihre Wirkung auf die Lesestrategiekompetenz und damit auf die Lesekompetenz der Kinder entfalten und wie die Kinder in diesen Programmen instruiert werden sollten, werden Prozessdaten zum gemeinsamen Lesen benötigt. Da diese Daten fehlen, ist bislang ungeklärt, wie Grundschülerinnen und Grundschüler ohne explizite Instruktion für ein bestimmtes Vorgehen gemeinsam einen Text bearbeiten, insbesondere ob sie dabei Lesestrategien anwenden, um ein (vertieftes) Verständnis für den Text zu erarbeiten. Darauf aufbauend ist zudem unklar, inwiefern die verbale Anwendung von Lesestrategien in Kleingruppen in Quantität und Qualität durch eine peer-gestützte Leseförderung beeinflusst werden kann.

Eine Möglichkeit zur Beantwortung dieser Fragen ist die handlungsnahe Erfassung der Anwendung von Lesestrategien über Beobachtungsdaten. Die Anwendung von Lesestrategien in Paaren oder Kleingruppen bietet die Chance, dass die kognitive Anwendung von Lesestrategien durch die Kooperation und Kommunikation in der Gruppe verbalisiert und damit sichtbar und beobachtbar wird. Mithilfe von Audio- oder Videoaufnahmen können diese Prozesse möglichst genau erfasst und intensiv ausgewertet werden (siehe z. B. Schünemann et al. 2017). In der vorliegenden Studie wurde daher die Zusammenarbeit von Kleingruppen bei der Arbeit an einem Text videographiert. Ziel der vorliegenden Studie ist es, den Einsatz von Lesestrategien in Kleingruppen von Grundschülerinnen und Grundschülern bei der Bearbeitung einer unstrukturierten Leseaufgabe zu evaluieren. Die Kinder erhielten daher nur die Aufgabenstellung, einen Text gemeinsam zu lesen und sicherzustellen, dass alle Gruppenmitglieder den Inhalt verstehen. Anhand der Videodaten soll (1) untersucht werden, ob Grundschülerinnen und Grundschüler der 3. Klasse beim kooperativen Lesen eines Textes spontan Lesestrategien einsetzen, (2) wie häufig die untersuchten Strategien über die drei Messzeitpunkte eingesetzt werden und ob Kinder, die an einer reziproken Leseförderung im Unterricht teilgenommen haben, im Vergleich zu Kindern einer untrainierten Unterrichtskontrollgruppe während einer Gruppenaufgabe mehr Strategien einsetzen, sowie (3) auf welchem Niveau die Qualität der Strategieanwendung zu drei Messzeitpunkten zu bewerten ist und ob sich Trainingseffekte auf das Qualitätsniveau zeigen.

## Methode

### Stichprobe

Die Untersuchungsstichprobe bestand aus 21 3. Klassen, die zufällig der Interventionsgruppe (IG; 11 Klassen) und der Unterrichtskontrollgruppe^1^ (KG) zugeordnet wurden. Es wurden nur Daten von Kindern eingeschlossen, für die eine Einverständniserklärung der Eltern für das Anfertigen von Videoaufnahmen vorlag. Die IG bestand aus *n* = 133 Kindern (*n* = 64 Mädchen, *n* = 68 Jungen, *n* = 1 fehlende Angabe) und die KG aus *n* = 136 Kindern (*n* = 63 Mädchen, *n* = 71 Jungen, *n* = 2 fehlende Angaben). Unabhängige T‑Tests ergaben keine signifikanten Unterschiede zwischen den Bedingungen hinsichtlich ihrer Lesefähigkeit im Lesetest *Ein Leseverständnistest für Erst- bis Siebtklässler – Version II* (ELFE II; Lenhard et al. 2018) im Prätest (*t*(261) = 0,364, *p* = 0,716). Alle teilnehmenden Schulen befanden sich in und in der Umgebung einer mittelgroßen Stadt in Deutschland.

### Leseförderung

Die IG wurde mit dem Lesetraining „Raffi und seine Freunde“ unterrichtet, einem Lesetraining konzipiert für die Grundschule, für das erste Wirksamkeitsbelege bereits vorliegen (Völlinger et al. 2018b). Den Mittelpunkt der Förderung bildet die Leseratte mit dem Namen „Raffi“, die gemeinsam mit ihren Freunden verschiedene Lesestrategien erlernt. Das Programm basiert auf verschiedenen evidenzbasierten Methoden zur Verbesserung der Lesekompetenz (Cooperative Integrated Reading and Composition [CIRC], Stevens et al. 1987; Reziprokes Lehren, Palincsar und Brown 1984; Peer-assisted learning strategies [PALS], Fuchs und Fuchs 2000) und sieht die Förderung der Leseflüssigkeit durch gemeinsames Lesen und des Leseverstehens durch die Vermittlung der Lesestrategien *Klären, Fragen *und *Vorhersagen* in leistungsheterogenen Kleingruppen vor.

Die Umsetzung der Leseförderung erfolgte in ganzen Klassen während des regulären Unterrichts durch geschulte studentische Hilfskräfte unter Anwesenheit der regulären Lehrkraft. Die Förderung fand für 20 Trainingsstunden zweimal wöchentlich für je eine Schulstunde (45 min) über einen Zeitraum von 10 Wochen statt. Die Leseförderung wurde regelmäßig supervidiert. Der genaue Ablauf der Förderung war in einem Manual detailliert vorgegeben. In den ersten beiden Sitzungen lernten die Kinder die Leseratte „Raffi“ und die Lesetrainerin bzw. den Lesetrainer kennen und es wurden Übungen zur Verbesserung der kooperativen Zusammenarbeit durchgeführt. Anschließend lernten die Kinder in Partnerarbeit einen Text zu erlesen und sich nach der Methode des reziproken Lehrens gegenseitig Feedback dazu zu geben. Dabei lasen die Kinder abwechselnd je einen Textabschnitt ihrer Lesepartnerin bzw. ihrem Lesepartner laut vor und wechselten sich somit in der Rolle des Tutors und des Lernenden ab. Die Partner gaben sich mithilfe eines entsprechenden Arbeitsblattes eine Rückmeldung bezogen auf fehlerfreies Lesen und eine angemessene Betonung. Dazu wurde ein entsprechender Smiley angekreuzt. Die Kinder arbeiteten mit Sachtexten, die in vier Abschnitte (je ca. 100 Wörter) unterteilt waren und für Kinder spannende Themen wie Tiere oder Berufe behandelten.

#### Gruppeneinteilung

Während der Leseförderung arbeiteten die Kinder zunächst in Partnerarbeit und im späteren Verlauf in Kleingruppen von 4–5 Kindern zusammen. Die Gruppeneinteilung erfolgte heterogen in Bezug auf die Lesefähigkeit. Die Schülerinnen und Schüler wurden entsprechend ihrer Prätestergebnisse im Leseverständnis, erhoben mit dem ELFE II (Lenhard et al. 2018) in eine Rangordnung gebracht. Diese Rangordnung wurde anschließend in zwei Hälften unterteilt. Einem Kind aus der oberen Hälfte des Rankings mit einem sehr hohen Wert in der Lesefähigkeit wurde eine Lesepartnerin bzw. ein Lesepartner mit einer geringeren Lesefähigkeit aus der oberen Hälfte zugeteilt. Entsprechend wurden Paare aus der unteren Hälfte gebildet. Anschließend wurde einem Paar der oberen Hälfte einem Paar der unteren Hälfte zugeteilt, sodass sich Gruppen von 4–5 Kindern ergaben, die im klasseninternen Vergleich im Mittel ähnliche Werte aufwiesen. Um die praktische Umsetzung der Gruppen zu gewährleisten, wurde die Gruppenzusammensetzung anschließend mit der Klassenlehrkraft in Bezug auf das soziale Miteinander der Kinder besprochen und je nach Einschätzung angepasst. Alle Gruppen zeigten, auch nach der Anpassung, in einer Klasse im Durchschnitt eine ähnliche Lesefähigkeitsleistung.

#### Lesestrategien

Im Lesetraining „Raffi und seine Freunde“ wurden die drei Lesestrategien *Klären, Fragen *und *Vorhersagen *vermittelt. Die Lesetrainerin bzw. der Lesetrainer modellierte zunächst die Anwendung jeder Strategie auf einen Text. Anschließend erhielten die Kinder die Aufgabe, in Partnerarbeit einen Text nach der Methode des reziproken Lesens abschnittsweise zu erlesen und zusätzlich für jeden Abschnitt die Lesestrategie anzuwenden. Das zuhörende Kind notierte dafür jeweils auf dem Feedbackbogen die Anwendung und gab dem lesenden Kind ein Feedback zur Leseleistung. Um den Gebrauch aller Strategie zu üben und damit die Anwendung zu verinnerlichen, wurde jede Lesestrategie zunächst einzeln in 2–3 Trainingsstunden implementiert.

##### *Klären*

Als erste Lesestrategie führte die Lesetrainerin bzw. der Lesetrainer in der 5. Trainingssitzung die Lesestrategie *Klären *ein. Bei dieser Strategie lernten die Kinder, unbekannte Wörter in einem Text zu identifizieren. Anschließend übten die Kinder, wie sie sich die Wortbedeutung selbstständig erschließen können (Wort genau anschauen, Satz und Zusammenhang noch einmal lesen, Partnerin bzw. Partner fragen, die Lesetrainer bzw. den Lesetrainer fragen).

##### *Fragen*

In der 7. Trainingssitzung erfolgte die Instruktion der Strategie *Fragen*. Die Schülerinnen und Schüler lernten, Fragen zu einem Textabschnitt zu stellen und die Fragenqualität der Lesepartnerin bzw. des Lesepartners zu bewerten. Dabei übten die Kinder, verschiedene Arten von Fragen zu stellen, die zu einem besseren Verständnis des Textes beitragen (siehe Fragenarten unter 4.4).

##### *Vorhersagen*

Ab der 10. Trainingssitzung lernten die Kinder die Strategie *Vorhersagen *anzuwenden. Die Schülerinnen und Schüler lasen dazu einen Textabschnitt und bekamen die Aufgabe, zu überlegen, wie der Text weitergehen könnte. Anschließend beurteilte die Lesepartnerin bzw. der Lesepartner, ob die Vorhersage wahrscheinlich war.

Ab der 12. Trainingssitzung wendeten die Kinder alle drei Lesestrategien in Partnerarbeit auf den Text an. Mit Beginn der 13. Trainingssitzungen arbeiteten die Kinder in Kleingruppen (4–5 Kinder). Dazu las ein Kind einen Textabschnitt vor und ein anderes Kind übernahm die Rolle des Tutors und gab mithilfe des bekannten Arbeitsblattes eine Rückmeldung zum Lesen und der Anwendung der Lesestrategien. Die anderen Kinder unterstützten die Leserin bzw. den Leser, indem sie den Text mitlasen und Tipps bezüglich der angewendeten Strategien gaben. Um den Transfer des Gelernten auf eine neue Textart zu gewährleisten, arbeiteten die Kinder ab der 16. Trainingssitzung mit narrativen Texten.

### Videobeobachtung

Wir verwendeten Videobeobachtungen, um den Strategiegebrauch während einer Gruppenaufgabe zu drei Messzeitpunkten (Prätest, Posttest und Follow-up) zu evaluieren. Die Kinder der IG sollten nicht mit den Kindern in einer Gruppe videographiert werden, mit denen sie im Lesetraining zusammengearbeitet hatten, um auszuschließen, dass es sich bei einer Verbesserung der Kinder der IG von vor zu nach dem Lesetraining um einen Gewöhnungseffekt handelte, der sich durch die höhere Vertrautheit in der Lesegruppe erklären ließe. Aus diesem Grund wurden die Kinder der IG für die Testsitzungen erneut, aber auch die Kinder der KG, in leistungsheterogene Kleingruppen eingeteilt (2–5 Kinder). Die Gruppeneinteilung erfolgte anhand der Prätestergebnisse in der Verlaufsdiagnostik sinnerfassenden Lesens (VSL; Walter 2013). Zur Berechnung der individuellen Leseleistung wurde der Mittelwert der zu fünf Baselinemessungen erhobenen Leistungen im VSL herangezogen. Die Gruppeneinteilung entsprach dem Vorgehen in 4.2.1. Die Gruppenzusammensetzungen blieben über alle drei Testsituationen gleich. In der IG wurden 35 Kleingruppen und in der KG 37 Kleingruppen videographiert. Die Schülerinnen und Schüler wurden instruiert, gemeinsam einen einseitigen Sachtext zu lesen und zu verstehen, um anschließend individuell ein Quiz zum Textinhalt auszufüllen. Weitere Instruktionen zur Vorgehensweise bei der gemeinsamen Textarbeit oder Materialien erhielten die Kinder nicht. Da die Schülerinnen und Schüler nicht explizit angewiesen wurden, die Strategien in der Testsituation zu verwenden, wurde somit die spontane Strategieverwendung in den Lesegruppen erfasst. Die Kleingruppen saßen bei den Videoaufnahmen jeweils an einem Gruppentisch. In der Tischmitte war ein Mikrofon platziert. Die Kamera wurde so aufgestellt, dass alle Kinder am Tisch zu sehen waren. In jeder Testung erhielten die Schülerinnen und Schüler 20 min Zeit für die Kleingruppenarbeit.

### Kodierschema zur Videoauswertung

Die Auswertung des Strategieeinsatzes der Schülerinnen und Schüler erfolgte anhand einer quantitativen Inhaltsanalyse (Mayring 2015). Mithilfe einer deduktiven Kategorienbildung auf Basis des Strategietrainings und bereits entwickelter Schemata (vgl. Schünemann et al. 2013) können so die beobachteten Aussagen reduziert und in quantitativ auswertbare Daten transformiert werden. Analyseebene war das Individuum. Als Analyseeinheit wurde eine inhaltlich geschlossene Aussage kodiert. Während des Kodierens erfolgten für jede Aussage zwei Entscheidungen: 1. Wurde eine Strategie angewendet? (Quantität); 2. Von welcher Qualität war dieser Strategieeinsatz?

Die Strategie *Klären *wurde kodiert, wenn in der Gruppe nach der Bedeutung eines Wortes gefragt wurde. Die Qualität des zu klärenden Wortes wurde einer von drei Kategorien zugeordnet:

- 0: das Wort hat nichts mit dem Text zu tun oder steht nicht im Text
- 1: ein Wort, das häufig in der deutschen Sprache vorkommt („und“, „Baum“, „Haus“) oder bereits im Text direkt oder indirekt erklärt wird
- 2: das Wort wird nicht bereits im Text erklärt; es handelt sich um ein eher unbekanntes Wort, das selten verwendet wird; Namen von Städten etc.

Eine *Frage *wurde kodiert, wenn sich diese auf den Textinhalt bezog. Diese wurde qualitativ in eine von sieben Kategorien eingeordnet:

- 0: eine Frage ohne Bezug zum Text; eine Frage, die nicht mit dem Text beantwortbar ist
- 1: Ja- oder Nein-Frage
- 2: Detailfrage, die sich auf einen nebensächlichen Aspekt bezieht und in der Formulierung aus dem Text übernommen wurde
- 3: Detailfrage, die sich auf einen nebensächlichen Aspekt bezieht, jedoch selbst formuliert wurde
- 4: Frage nach der Hauptidee des Absatzes und in der Formulierung aus dem Text übernommen wurde
- 5: Frage nach der Hauptidee, die selbst formuliert wurde
- 6: Frage nach Schlussfolgerungen, Vergleichen, Bewertung, Ursache-Wirkung

*Vorhersagen *wurden dann kodiert, wenn die Schülerinnen und Schüler eine Vermutung äußerten, wie der Text weitergehen könnte. Um zu überprüfen, wie gut die Strategie umgesetzt wurde, wurden die Aussagen einer von vier Kategorien zugeordnet:

- 0: kein Bezug zum Text; Wiedergabe des Textes, ohne eine Vorhersage zu formulieren
- 1: Vorhersage, mit vagem Bezug zum Text; „Fantasie-Erzählung“
- 2: eine zum Text passende Vorhersage, die jedoch eher einen allgemeinen Aspekt umfasst, der zum Thema passt
- 3: Vorhersage mit klarem Bezug zum Textabschnitt, die sehr wahrscheinlich ist

Die beobachtete Strategieanwendung wurde von zwei unabhängigen geschulten Kodierpersonen anhand eines ausführlichen Kodiermanuals bewertet (30 % Doppelkodierung zur Interraterkontrolle). Hierzu wurde das Programm* Interact* von Mangold International GmbH (1998–2014) verwendet. In die Auswertung der Quantität der eingesetzten Strategien gingen die Summen der einzelnen Kodierungen pro Schülerin oder Schüler ein. Um die Qualität der Strategieanwendung zu überprüfen, wurde bei mehrmaliger Nutzung einer Strategie der höchste erreichte Wert der jeweiligen Kategorie vergeben. Es wurden nur Beobachtungen kodiert, die sich in das angegebene Schema einordnen ließen. Die Kodierpersonen notierten zusätzlich, wenn die Kinder ein von den dargestellten Strategien abweichendes Vorgehen zeigten. Die Beobachterübereinstimmungen sind in Tab. 1 dargestellt. Da die Strategie Vorhersagen in einem sehr geringen Umfang angewendet wurde, ließ sich bei der Qualität der Anwendung der Strategie kein Wert der Übereinstimmung berechnen.

**Table 1:** 

Strategie	Quantität	Qualität
Klären	0,520	0,341
Fragen	0,642	0,807
Vorhersagen	1.000	/

### Datenanalyse

Zur Analyse der vorliegenden quantitativen Daten wurde das Programm IBM SPSS Statistics 28 verwendet. Zur Beantwortung der ersten Fragestellung, ob Schülerinnen und Schüler in der Testsituation Strategien einsetzen, werden deskriptive Statistiken zum Anteil der Schülerinnen und Schüler dargestellt, die in ihren Gruppen Strategien nutzten. Zur Beantwortung der zweiten Fragestellung nach der Häufigkeit der Anwendung für die Strategien *Fragen, Zusammenfassen* und *Vorhersagen* und der Veränderung über die Messzeitpunkte berechneten wir jeweils eine Varianzanalyse mit Messwiederholung. Zudem stellen wir die Entwicklung in der Qualität der Strategieanwendung anhand deskriptiver Werte dar und prüfen Gruppenunterschiede anhand von T‑Tests für unabhängige Stichproben.

## Ergebnisse

### Quantität der Strategienutzung

Die Auswertung der Videoaufnahmen zeigt, dass im Prätest 27,9 % der Schülerinnen und Schüler in ihren Gruppen mit Lesestrategien arbeiteten. Im Posttest waren es 44,2 % und im Follow-up-Test 38,0 %. Die Strategienutzung ist Tab. 2 zu entnehmen.

**Table 2:** 

	Prätest	Posttest	Follow-up-Test
Interventionsgruppe	30,1	48,9	47,4
Kontrollgruppe	26,1	40,1	23,9
Gesamt	27,9	44,2	38,0

**Figure 1:**
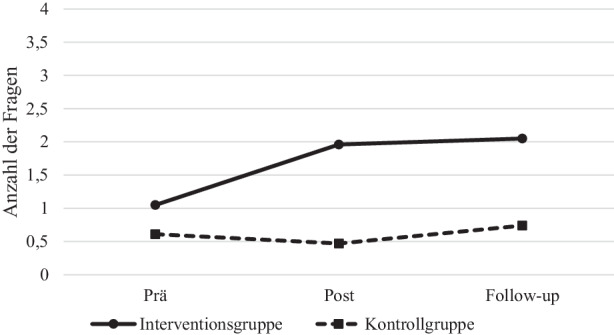

Die Berechnung von T‑Tests für abhängige Stichproben zeigen für den Vergleich von IG und KG, dass es keinen mittleren Unterschied in der Quantität des Strategieeinsatzes in den Kleingruppen beim Vortest gibt (*Klären, Fragen, Vorhersagen* alle *ps* > 0,05). Für die Anzahl der Klärungen gibt es zum Post- oder Follow-up-Test keinen statistisch bedeutsamen Unterschied zwischen den Bedingungen (*ps* > 0,05). Im Posttest und im Follow-up-Test wurden in der IG signifikant mehr Fragen gestellt als in der KG (Posttest: *T* (273) = 5,478, *p* < 0,001, *d* = 0,66; Follow-up: *T* (273) = 3,97, *p* < 0,001, *d* = 0,48) und mehr Vorhersagen getroffen (Posttest: *T* (273) = 2,71, *p* = 0,007, *d* = 0,32, Follow-up-Test: *T* (273) = 3,20, *p* = 0,002, *d* = 0,40).^2^ Varianzanalysen mit Messwiederholung für *Klären* ergeben für die Anzahl der geklärten unbekannten Wörter weder eine bedeutsame Entwicklung noch eine Interaktion mit der Bedingung (*ps* > 0,05). Für *Fragen* zeigt sich ein bedeutsamer Anstieg in der Anzahl der formulierten Fragen über die Zeit (*F* (2, 546) = 4,75, *p* = 0,009) und auch eine signifikante Interaktion von Messzeitpunkt und Bedingung (*F* (2, 546) = 4,41, *p* = 0,013) die eine stärkere Entwicklung in der IG impliziert (siehe Abb. 1). Auch die Anzahl der getroffenen Vorhersagen steigt über die Zeit deskriptiv an, verfehlt jedoch die Signifikanzgrenze (*p* = 0,052). Für die Interaktion von Messzeitpunkt und Bedingung und somit für eine unterschiedliche Entwicklung im Verlauf in IG und KG, ergibt sich ein statistisch bedeutsamer Wert (*F* (2, 546) = 4,88, *p* = 0,014). So steigt die Anzahl der getroffenen Vorhersagen in der IG stärker an.

Darüber hinaus beobachteten wir, ob die Schülerinnen und Schüler weitere Strategien oder Routinen bei der Textbearbeitung anwenden. Es zeigt sich jedoch, dass kaum andere Strategien auf die Texte angewendet wurden. In vereinzelten Gruppen versuchten die Schülerinnen und Schüler, wichtige Informationen im Text zu identifizieren. Dazu gingen sie unterschiedlich, jedoch kaum strategisch vor. So versuchten sie unsystematisch zentrale Informationen zu markieren (1 Gruppe) oder zu benennen (2 Gruppen) bzw. Inhalte aus dem Text im Gespräch zu wiederholen (1 Gruppe).

### Qualität der Strategienutzung

Für die Qualität der Strategien *Klären* und *Vorhersagen* ergibt sich kein bedeutsamer Unterschied zum Prätest zwischen den Bedingungen (*p* > 0,05; siehe auch Tab. 3). In der Qualität der gestellten *Fragen* ergeben sich für den Prätest Unterschiede zugunsten der IG (Prä: *T* (42) = 2,70, *p* = 0,010, *d* = 0,85). Für die Qualität der Klärungen zeigt sich kein Unterschied zwischen den Bedingungen im Post- oder Follow-up-Test (alle *ps* > 0,05). Im Posttest lag die Qualität der gestellten Fragen in der IG höher als in der KG (Post: *T* (93) = 3,53, *p* = 0,001, *d* = 0,74) während sich im Follow-up-Test ein bedeutsamer Unterschied zugunsten der KG zeigt (FU: *T* (86) = −2,41, *p* = 0,018). Weder im Post- noch im Follow-up-Test wurde die Strategie *Vorhersagen* in der KG eingesetzt. Aufgrund der Datenstruktur (kleine Substichproben und fehlende Varianz in den Daten) ist die Berechnung von Varianzanalysen mit Messwiederholung für die Qualität der Strategieanwendungen nicht möglich.

**Table 3:** 

Bedingung		*N*	Minimum	Maximum	*M*	*SD*
Interventions-gruppe	Klären Prä	4	1	2	1,25	0,50
	Klären Post	8	1	2	1,56	0,50
	Klären Follow-up	8	1	2	1,31	0,46
	Fragen Prä	29	0	6	2,79	1,78
	Fragen Post	57	0	6	2,63	1,41
	Fragen Follow-up	63	0	6	2,68	1,69
	Vorhersagen Prä	0	–	–	–	–
	Vorhersagen Post	10	2	3	2,30	0,48
	Vorhersagen Follow-up	11	2	3	2,55	0,52
Unterrichts-kontrollgruppe	Klären Prä	4	1	2	1,43	0,51
	Klären Post	6	1	2	1,25	0,42
	Klären Follow-up	4	1	2	1,50	0,58
	Fragen Prä	15	0	2	1,47	0,92
	Fragen Post	38	0	3	1,66	1,17
	Fragen Follow-up	25	1	6	3,64	1,66
	Vorhersagen Prä	2	0	1	0,50	0,71
	Vorhersagen Post	0	–	–	–	–
	Vorhersagen Follow-up	0	–	–	–	–
Gesamt	Klären Prä	8	1	2	1,34	0,48
	Klären Post	14	1	2	1,43	0,48
	Klären Follow-up	12	1	2	1,38	0,48
	Fragen Prä	44	0	6	2,34	1,66
	Fragen Post	95	0	6	2,24	1,37
	Fragen Follow-up	89	0	6	2,98	1,73
	Vorhersagen Prä	2	0	1	0,50	0,71
	Vorhersagen Post	10	2	3	2,30	0,48
	Vorhersagen Follow-up	12	2	3	2,50	0,52

## Diskussion

Die Auswertung der Videoaufnahmen zeigt, dass für einen großen Teil der Schülerinnen und Schüler der Einsatz von Lesestrategien nicht die Routine der Wahl bei der gemeinsamen Arbeit an einem Text war: So setzten weniger als die Hälfte der Kinder die Strategien *Fragen, Klären* oder *Vorhersagen *bei der gemeinsamen Erarbeitung eines Lesetextes ein. Vergleicht man die Schülerinnen und Schüler der IG und KG, so zeigt sich, dass es zwar keinen Unterschied in der Quantität des Strategieeinsatzes in den Kleingruppen zum Prätest gibt, die Kinder in der IG aber im Posttest und im Follow-up-Test die Lesestrategien *Fragen *und *Vorhersagen *häufiger einsetzten als die Schülerinnen und Schüler der KG. Die Strategie *Vorhersagen* wurde in der KG nicht eingesetzt. Ein Trainingseffekt ist daher insbesondere für *Vorhersagen* zu sehen: Die Schülerinnen und Schüler erstellten im Prätest kaum Vorhersagen. In der Trainingsgruppe zeigt sich jedoch ein Anstieg in den formulierten Vorhersagen im Post- und Follow-up-Test, der sich in der KG nicht zeigt, wobei die Anzahl der gestellten Vorhersagen auch in der IG gering war.

Die Strategie *Fragen* wurde am meisten eingesetzt. Damit scheint diese Strategie sich häufiger im Strategierepertoire von Schülerinnen und Schülern zu finden bzw. sich einfacher in dieses zu integrieren, sodass Produktions- und Nutzungsdefizite bei dieser Strategie seltener auftreten. Allerdings ist die Qualität der gestellten Fragen nicht auf einem hohen Niveau einzuordnen. Im Mittel erzielten die Schülerinnen und Schüler beider Gruppen zum Follow-up-Test einen Wert von 2,98 (bei einem möglichen Wert zwischen 0 und 6). Das Ergebnismuster für die Qualität der gestellten Fragen ist insgesamt schwierig zu interpretieren. Auffällig ist, dass der mittlere Wert für die Qualität der gestellten Fragen im Follow-up-Test in der KG mit 3,64 höher ausfällt als in der IG mit 2,68. In der IG ist zudem der Wert für die Qualität der eingesetzten Fragen im Prätest höher als im Post- und Follow-up-Test. Diese Inkonsistenzen sind eventuell auch darauf zurückzuführen, dass die Stichprobe, die für die Bewertung der Qualität des Strategieeinsatzes herangezogen werden kann, klein ausfällt. Weitere Studien mit größeren Stichproben wären daher hilfreich, um an dieser Stelle zu tragfähigeren Aussagen zu kommen.

Während die Anzahl der Klärungen sich über die Messzeitpunkte leicht verringert, ergibt sich über beide Gruppen keine Veränderung in der Qualität der Klärungen unbekannter Wörter oder Begriffe. Zwischen den Bedingungen bestehen zudem keine bedeutsamen Unterschiede. Ob den Schülerinnen und Schülern in den Texten alle Wörter bekannt waren und sie die Strategie daher selten einsetzten, bleibt offen. Zu vermuten ist jedoch, dass diese Strategie noch nicht ausreichend im Strategierepertoire der Schülerinnen und Schüler verankert werden konnte und daher seltener eingesetzt wurde. Die errechnete Beobachterübereinstimmung fällt für die Qualität der Klärungen am niedrigsten aus. Das Kodiermanual sollte daher für weitere Arbeiten eine Überprüfung unterzogen werden. Die Übereinstimmung bezieht sich auf eine geringe Anzahl von kodierten Werten, was ebenso die Güte beeinflusst haben kann.

Beobachtet man eine beispielhafte Gruppensituation (im ergänzenden online Material ist ein Transkript verfügbar) wird deutlich, wie die Schülerinnen und Schüler in einer Gruppe mit den Lesestrategien am Text arbeiten können. Sichtbar wird aber auch, dass die vorgegebenen Strategien, wenn sie verwendet werden, nicht automatisch zu einer starken Elaboration des Textes führen: Die Kinder nutzen die Routine, arbeiten dabei jedoch wenig elaboriert mit dem Text. Die intensivste Auseinandersetzung erfolgt an der Textoberfläche mit Fokus auf korrektes und flüssiges Vorlesen.

Das in dieser Studie eingesetzte Förderprogramm entspricht den Empfehlungen zur Gestaltung wirksamer Förderprogramme (Artelt et al. 2007), basiert auf evidenzbasierten Methoden (Fuchs und Fuchs 2000, Palincsar und Brown 1984, Stevens et al. 1987) und zeigte in einer ersten Evaluationsstudie positive Effekte auf die Leseleistungen von trainierten Kindern (Völlinger et al. 2018b). Allerdings ergibt sich in der vorliegenden Untersuchung, dass nach dem Training in einer Lesesituation analog zur Förderung (gemeinsames Lesen eines Textes in einer Kleingruppe) kein vollständiger Situationstransfer der erlernten Strategien und Routinen stattfindet. Die Testsituation unterscheidet sich vom gemeinsamen Lesen in der Förderung durch eine veränderte Gruppenzusammensetzung. Zudem haben die Kinder kein unterstützendes Material zur Verfügung, sondern arbeiten ausschließlich mit dem Text ohne weitere Hilfestellungen oder Prompts zur Anwendung der Lesestrategien und ohne die explizite Instruktion, Strategien anzuwenden. Es handelt sich also um eine Anwendungssituation, die einen Transfer der in der Förderung eingeübten Kompetenzen erfordert (Perkins und Salomon 1989).

Es sind verschiedene Erklärungsansätze für den geringen Strategieeinsatz möglich. So könnte es sein, dass 1.) der notwendige Transfer in einen ähnlichen, dennoch anderen Kontext (Barnett und Ceci 2002) den beobachteten Kindern nicht auf einem hohen Niveau gelingt, weil das Training nicht ausreichend war in Umfang und Intensität, um die neu erlernten Strategien im Repertoire der Schülerinnen und Schüler so zu verankern, dass sie in der veränderten Aufgabensituation darauf zurückgreifen. Dann wäre es sinnvoll, das Training weiter fortzuführen und zusätzlich den Aufgaben- und Situationstransfer durch spezielle Inhalte im Training zu unterstützen.

Es könnte auch sein, dass 2.) die Schülerinnen und Schüler keine Notwendigkeit sahen, die Strategien anzuwenden, weil sie das Textmaterial als leicht einstuften und keine für sie offensichtlichen Verständnisschwierigkeiten hatten. Ob die Schülerinnen und Schüler die Texte vollständig verstanden haben, ist anhand der vorliegenden Daten nicht zu beantworten.^3^ So bleibt die Frage offen, ob die Schülerinnen und Schüler, welche verstärkt Lesestrategien anwendeten, auch ein besseres Verständnis für die gelesenen Texte entwickelten, als die Kinder in den Kleingruppen, in denen keine Lesestrategieanwendung stattfand.

Betrachtet man gezielt die Interaktion der Kinder in einer Gruppe (sichtbar im Transkript enthalten im Online-Material) wird zudem 3.) deutlich, dass außer der Anwendung von Lesestrategien bei der gemeinsamen Textarbeit noch weitere Anforderungen an die Schülerinnen und Schüler bestehen: So erfordert die Zusammenarbeit auch gemeinsame Regulation in der Gruppe (*socially shared regulation of learning*; siehe Hogenkamp et al. 2021; Volet et al. 2009) sowie metakognitive Aktivität der Schülerinnen und Schüler. Diese Merkmale werden beispielsweise dann sichtbar, wenn ein Kind formuliert, dass man noch eine Frage und Vorhersage zum Absatz formulieren müsse oder dass am Ende eines Textes keine weitere Vorhersage notwendig sei. Solche Merkmale der Gruppenregulation sollten in zukünftigen Untersuchungen ebenfalls systematisch beobachtet und analysiert werden, da beispielsweise die direkte Regulation einer Gruppe durch eine Person (*other-regulation*) auch zu negativen Effekten auf Lernergebnisse führen kann (Rogat und Adams-Wiggins 2014). Damit einher geht die Frage, ob die Zusammenarbeit in den Gruppen durch fehlende kooperative Kompetenzen beeinflusst wurde. So ergeben sich auch Hinweise darauf, dass die Kinder nicht immer wertschätzend miteinander umgingen (siehe Transkript).

Informationen dazu, ob und wie Kinder in der Zusammenarbeit Strategien auf Texte anwenden, sind bedeutsam für die weitere Entwicklung von Trainingsprogrammen sowie die Arbeit in der Praxis. Insbesondere für die wirksame Implementation von Maßnahmen in den Unterricht und die notwendige Dissemination evidenzbasierter Konzepte, die aktuell noch auf einem zu niedrigen Niveau einzuordnen ist (Bremerich-Vos et al. 2017), sind diese Informationen relevant. Nur wenn gesichertes Wissen über Lernprozesse im Rahmen von Methoden besteht, kann dieses auch angemessen aufbereitet an Lehrkräfte weitergegeben werden. Dazu sind neben den Informationen zur Wirksamkeit von Lesestrategietrainings (Mayer und Marks 2019; Slavin et al. 2009) auch Informationen zur Wirkweise der Programme notwendig, die es möglich machen, überzeugende und konkrete Hilfestellungen für die Integration solcher Programme in den regulären Unterricht zu formulieren, um die Implementation evidenzbasierter Methoden zur Förderung von Strategieanwendung und Leseverständnis dadurch zu unterstützen.

### Supplementary Information





## Anhang

**Table 4:**

Bedingung		*N*	Minimum	Maximum	*M*	*SD*
Interventions-gruppe	Klären Prä	133	0	2	0,16	0,42
	Klären Post	133	0	5	0,23	0,64
	Klären Follow-up	133	0	3	0,21	0,55
	Fragen Prä	133	0	12	1,05	2,74
	Fragen Post	133	0	9	1,96	3,06
	Fragen Follow-up	133	0	11	2,05	3,08
	Vorhersagen Prä	133	0	0	–	–
	Vorhersagen Post	133	0	4	0,14	0,63
	Vorhersagen Follow-up	133	0	3	0,14	0,50
Unterrichts-kontrollgruppe	Klären Prä	142	0	7	0,28	0,81
	Klären Post	142	0	4	0,25	0,61
	Klären Follow-up	142	0	3	0,15	0,44
	Fragen Prä	142	0	14	0,61	2,47
	Fragen Post	142	0	5	0,47	1,04
	Fragen Follow-up	142	0	13	0,74	2,38
	Vorhersagen Prä	142	0	1	0,01	0,12
	Vorhersagen Post	142	0	0	–	–
	Vorhersagen Follow-up	142	0	0	–	–
Gesamt	Klären Prä	276	0	7	0,22	0,65
	Klären Post	276	0	5	0,24	0,62
	Klären Follow-up	276	0	3	0,18	0,50
	Fragen Prä	276	0	14	0,82	2,60
	Fragen Post	276	0	9	1,19	2,37
	Fragen Follow-up	276	0	13	1,38	2,81
	Vorhersagen Prä	276	0	1	0,01	0,09
	Vorhersagen Post	276	0	4	0,07	0,44
	Vorhersagen Follow-up	276	0	3	0,07	0,37

## Footnotes

1 Die KG war in der Untersuchung als Wartelistenkontrollgruppe geplant. Aufgrund der Schulschließungen im Rahmen der Covid-19 Pandemie konnte eine Förderung in der KG jedoch nicht mehr durchgeführt werden.

2 Deskriptive Statistiken zum Strategieeinsatz in den Bedingungen zu den drei Messzeitpunkten sind im ergänzenden Onlinematerial enthalten.

3 Ein am Ende der Stunde eingesetzter Test zum Textinhalt, erzielte Deckeneffekte und war daher nicht zur Evaluation des Textverständnisses nutzbar.
