# Supplementary material for: Quantity and quality of elementary school students’ strategy use in reciprocal reading groups
Source: ZfG. 2022 May 24;15(2):361–78. [Article in German] doi: 10.1007/s42278-022-00142-1 (PMC9127815; doi:10.1007/s42278-022-00142-1)
Supplement: Supplementary file 1 [file 42278_2022_142_MOESM1_ESM.docx]

**ANHANG**

Das folgende Transkript (transkribiert nach Kuckartz et al. 2008) einer gemeinsamen Textarbeit der Kinder dient zur Verdeutlichung, wie die Kinder in den Gruppen, in denen Strategien eingesetzt wurden, interagierten. Es handelt sich um eine Kleingruppe (4 Kinder) der IG, die zum Follow-Up Zeitpunkt aufgenommen wurde.

| Transkript Anfang:  **K3:** M. fang an.  **K2:** (liest) Das Leben der Elefanten. Wir befinden uns in Afrika. Nähe (,) nahe (K3: Nahe) eines Flusses sieht man große graue Steine, die Biene .. (K3: die Beine) die Beine haben. Eine Elefantenfamilie schaukelt Richtung Ufer. Es ist Hochsommer und die meisten Wasserstellen haben bereits kein Wasser mehr, weil es so heiß ist. Deswegen laufen dei (K3: die) Elefanten eine sehr lange Weg (K3: einen sehr langen) Weg, um hier an den Fluss zu bekommen (K3: zu kommen) zu kommen. Sie trinken und duschen in dem Wasser der (,) des Flusses.  **K3:** Jetzt Frage.  **K2:** Also … warum haben die kein Wasser mehr?  **K3:** Weil es so heiß ist. Jetzt Vorhersage.  **K1:** Weil es in Afrika zu heiß ist.  **K2:** Okay, Vorhersage. Es könnte passieren, dass… (K3: ich weiß) wie die Elefanten Babys bekommen.  **K3:** Okay, jetzt bin ich. Afrikanische Elefanten sind die größten und schwersten Tiere, die an Land leben. Ein ausgewachsenes Weibl (,) Weibchen bringt .. (K2: dreitausend) ich weiß … dreitausend Kilogramm auf die Waage. Nämlich Elefanten werden Bullen genannt und können sogar siebentausend Kilogramm wiegen. So viel wiegen fünf Schuhklassen mit ihren Lehrern zusammen. Trotzdem ihrer Masse sind die aber nicht grob und tallpatschig.    **K2:** Tollpatisch .. du hast tallpatschig (-)  **K3:** Unten am Fluss läuft auch ein kleines Elefantenjunges. Obwohl die älteren Elefanten nur Augen fürs Wasser haben, rempeln und treten sie keine Kleinen nie. Ich mach erstmal Vorhersage.  **K2:** Sie das Kleine nie.  **K3:** Okay.  **K2:** Erst Frage.  **K3:** Nein, ich mache erst Vorhersage. Vielleicht wird ja gesagt, warum sie Bullen genannt werden. Und die Frage ist (-)    **K4:** Bullen, nicht Bullen, sondern Bullen.  **K3:** Ja … Wie werden männliche Elefanten genannt?  **K2:** Bollen.  **K3:** Falsch. Nicht Bollen.  **K1:** ⸢Bullen.  **K4:** ⸤Bullen.  **K2:** Ja, Bullen.  **K3:** Und die Vorhersage ist, warum sie so genannt werden.  **K2:** Du bist (guckt zu K1).  **K3:** Du bist (zeigt auf K1).  **K1:** Fast zwölf Jahre kümmern sich die erwachsenen Elefanten in (-) interesiv (K3: intensiv) intensiv un den Nachwuchs (-)    **K2:** Falsch un hast du gesagt, um, um den Nachwuchs.  **K1:** Ich habe gesagt um… um den Nachwuchsch.  **K2:** ⸢Nachwuchs.  **K4:** ⸤Nachwuchs.  **K3:** Du hast Nachwuchsch gesagt.  **K1:** Ja (`). Sie bringen dem kleinen Elefanten bei wie er mit dem Rüssel Gras zupfen kann. Gras ist die Hauptnahrung der Elefanten. Elefanten benutzen ihr Rüssel zum Atmen, zum Riechen und zum Greifen. Trinken und Essen können sie damit jedoch nicht.  **K2:** Frage.  **K3:** Ich weiß eine Frage. Kann ich für dich?  **K1:** Nee ich will.  **K3:** Hä, eigentlich machen wir es ganz falsch. Eigentlich soll der andere aussuchen, weil es geht ja darum, ob wir den Text verstanden haben.  **K1:** Wie lange kümmern sich die Elefantenen .. äh .. eltern um Nachwuchs?  **K3:** Hä, was?  **K2:** Zwölf Jahre.  **K1:** Ja. Vorhersage.  **K2:** Such dir jemanden aus. Such dir doch jemanden aus.  **K3:** Nee, sie macht jetzt einfach keine.  **K1:** Doch, E., mach du.  **K3:** Nee, ich wollte Frage.  **K1:** Wer macht jetzt eine Vorhersage?  **K2:** Keiner. T., du bist (zeigt auf K4).  **K4:** Was?  **K1:** ⸢Du bist.  **K2:** ⸤Du bist.  **K2:** Der letzte Abschnitt.    **K4:** Wenn sich ein Mensch Dinge gut merken kann, sagt man oft er habe ein Elefanten (-) (K3: Elefantengedächtnis) gedächtnis. Das kommt daher, dass Elefanten sich Sachen sehr gut merken können und ein gutes .. Gedächtnis haben. So .. wissen die .. älteren Elefanten den schnell (-) (K3: schnellsten) sten Weg zu dem Fluss und führen ihre Familie zum Meer dorthin.  **K2:** Fertig.  **K3:** Nee, Frage, Vorhersage.  **K2:** Achso.  **K3:** Frage.  **K2:** T., wer macht die Frage?  **K4:** E.  **K3:** Ähhhh warte. … Wann führ, ähm, führen die älteren Elefanten, ähm, also, ähm die Familie dahin?  **K2:** Im Sommer. Dorthin. … Wer sagt Vorhersage?  **K4:** Ähhhm. Wer will?  **K2:** Mir ist egal.  **K3:** Du kannst doch M.  **K2:** Ich will.  **K3:** Eigentlich brauchen wir keine Vorhersage, weil wir haben ja den ganzen Text, gibt mehr keine.  **K2:** Sollen wir nochmal? Dann können wir uns alles viel mehr merken.  **K1:** Jeder allein im Kopf.  **K3:** Ja okay, den Text lesen wir alleine. Wer liest denn hier so laut?  (Alle lesen leise den Text. K1 und K4 schauen die meiste Zeit am Anfang zu den anderen Tischen.)  Transkript Ende. | ***Frage***  ***Vorhersage***  ***Vorhersage***  ***Frage***  ***Wiederholung der Vorhersage***  ***Frage***  ***Frage*** |
| --- | --- |

*Anmerkung:* ⸤⸢: gleichzeitiges Sprechen; langen: auffällige Betonung; Ufer: gedehnt; (liest): nichtsprachliche Vorgänge (Kallmeyer und Schütze 1976; zitiert nach Kuckartz et al. 2008)

Im Transkript ist sichtbar, dass die Lesegruppe die reziproke Arbeitsweise aus dem Förderprogramm in großen Teilen übernommen hat. In dieser Sequenz haben die Schülerinnen und Schüler keine unbekannten Wörter oder für sie unverständliche Sätze geklärt. Die Kinder haben die Strategien *Fragen* und *Vorhersagen* jeweils passend eingesetzt. Alle vier Kinder in dieser Gruppe haben sich bei der Textarbeit beteiligt. Die formulierten Vorhersagen haben vagen Textbezug (Kategorie 1) bzw. sind eher allgemein (Kategorie 2). Die formulierten Fragen beinhalten einmal eine Hauptidee (Kategorie 4), erfragen aber sonst Detailinformationen (Kategorie 2).

Kuckartz, U., Dresing, T., Rädiker, S., & Stefer, K. (2008). Qualitative Evaluation. Der Einstieg in die Praxis. *VS Verl. für Sozialwissenschaften*, *2,* https://doi.org/10.1007/978-3-531- 91083-3
